# Development of active chitosan film containing bacterial cellulose nanofibers and silver nanoparticles for bread packaging

**DOI:** 10.1002/fsn3.4424

**Published:** 2024-08-23

**Authors:** Jalal Sadeghizadeh Yazdi, Mahdieh Salari, Mohammad Hasan Ehrampoush, Mehrasa Bakouei

**Affiliations:** ^1^ Department of Food Science and Technology, School of Public Health Shahid Sadoughi University of Medical Sciences Yazd Iran; ^2^ Research Center for Food Hygiene and Safety, School of Public Health Shahid Sadoughi University of Medical Sciences Yazd Iran; ^3^ Department of Food Science and Technology, Faculty of Agriculture University of Tabriz Tabriz Iran; ^4^ Department of Environmental Health Engineering, School of Public Health Shahid Sadoughi University of Medical Sciences Yazd Iran

**Keywords:** active packaging, Ag nanoparticle, cellulose nanofibers, chitosan, wheat bread

## Abstract

The objective was to develop an active chitosan‐based coating and to evaluate its effect on the shelf life and microbial safety of bread. Bacterial cellulose nanofibers (BCNF) and various levels (0.5%, 1%, and 2%) of silver nanoparticles (AgNPs) were in the chitosan (CS) film. Characterization of films was determined by analyzing WVP, ultraviolet barrier, and opacity as well as FTIR, XRD, DSC, TGA, and SEM. The water vapor permeability (WVP) of CS was remarkably (*p* < .05) decreased from 3.75 × 10^−10^ to 0.85 × 10^−10^ g/smPa when filled with BCNF and 2% AgNPs. Thermal and structural properties were enhanced in nanoparticle‐included films. Applying CS/BCNF/AgNPs coatings for bread samples demonstrated a significant improvement in moisture retention and a decrease in the hardness (from 10.2 to 7.05 N for CS and CS/BCNF/1% AgNPs coated samples, respectively). Moreover, microbial shelf life of bread sample increased from 5 to 38 days after packaging with CS/BCNF/2% AgNPs film. After a storage period of 15 days at 25°C, no fungal growth was detected in bread samples which were coated with nanocomposite suspensions containing 1% and 2% AgNPs. However, at the same condition, yeast and mold counts was 7.91 log CFU/g for control sample. In conclusion, the CS/BCNF/2% AgNPs film might have the potential for use as active packaging of bread.

## Graphical abstract

Barrier, thermal, and structural properties of chitosan film were greatly enhanced by the incorporation of BCNF and AgNPs. Coated bread showed higher water activity, moisture content, and softness compared to the control one. AgNPs improved antifungal effects of chitosan‐based films, so nanocomposite films can be applied to extend the shelf life of baked products

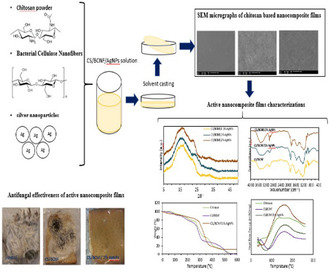

## 1 INTRODUCTION

Bread's widespread consumption globally is attributed to its high nutritional value and sensorial characteristics (Mihaly Cozmuta et al., 2015). The primary factors leading to the degradation of bread are microbial spoilage and staling, which causes vast food waste (estimated at 5%–10% of global bread production) and significant economic losses around the world (Melini & Melini, 2018; Srisa & Harnkarnsujarit, 2020). At room temperature, unpreserved bread typically maintains a relatively short shelf life, lasting around 2–4 days (Hosseiniyeh et al., 2023), limiting its marketability. Nowadays, the production, purchasing, and consumption of bread are modified, and it is expected to enhance the shelf life of this product (Pasqualone, 2019). Microbial contamination in bakery products stems from bacteria, yeasts, and fungi, with fungi being the most prevalent spoilers. They are responsible for the off‐flavor, allergic compounds, and mycotoxin. Fungal contamination of bread can occur during various stages, including cooling, slicing, packaging, and storage. It will not happen in baking processes because the baking temperatures are enough to inactivate molds or mold spores (Pateras, 2007). The major species involved are *Penicillium*, *Aspergillus*, *Mucor*, *Endomyces*, *Monilia*, *Cladosporium*, *Alternaria*, *Rhizopus*, and *Fusarium* (Araújo et al., 2023). As mentioned by Moe et al. (2023), *Aspergillus niger* and *Penicillium* spp. account for 60% of spoilage in bakery products. Using chemical preservatives, such as benzoic acid, sorbic acid, dehydroacetic acid, is a standard approach to prevent the growth of fungi in bread. However, in recent years, the demand for natural food products has increased (Z. Bao et al., 2023) and it is required to find a substitute for these chemicals. As an answer, active packaging represents an encouraging method to enhance both the shelf life and microbiological safety of bread. For instance, gelatin film containing beeswax and DATEM (diacetyl tartaric ester monoglycerides) (Mohammadi et al., 2023) are emerging as effective options. Incorporating nano silica and clove essential oil into poly(hydroxybutyrate) film present another avenue for active packaging solutions (Mittal et al., 2023).

In recent years, the buildup of plastic waste has become a major environmental issue worldwide as this material is nonbiodegradable. Therefore, eco‐friendly packaging is a preferable alternative to synthetic polymers. This is due to its numerous advantages, including reduced carbon emission throughout the composting and manufacturing, biocompatibility, and absence of other environmentally adverse effects (Duan et al., 2021; Kumar et al., 2020).

Chitosan is a natural edible polymer that is biodegradable and is obtained from crustacean exoskeletons through the alkaline N‐deacetylation process of chitin (Zagloul et al., 2024). Incorporating CS in the production of composite films has demonstrated advantageous characteristics, including biocompatibility, biodegradability, nontoxicity, exceptional thermal stability, capability for metal chelation, outstanding ability to form films, along with antioxidant, fungistat, and bacteriostat characteristics (Khanzada et al., 2023). Due to its insolubility in water, CS proves to be well suited to be used in active packaging with minimal swelling index and water permeability. Since chitosan films exhibit relatively poor mechanical properties, they must be modified by reinforcing nanofillers to create nanocomposite films. In this regard, previous studies have proven that the mechanical and physicochemical characteristics of chitosan‐based films can be enhanced by adding reinforcing nanofillers such as alumina (Al_2_O_3_) (Baskar et al., 2024), boehmite (Meera & Ramesan, 2023a), hydroxyapatite (Ramesan et al., 2024), and zein‐loaded cinnamaldehyde (Cin@ZN) (Wang et al., 2024) nanoparticles.

Cellulose synthesized by bacteria (mainly *Komagataeibacter xylinus*) possesses an identical chemical structure to that found in plants with the formula (C_6_H_10_O_5_) n (Sá et al., 2020). However, bacterial cellulose (BC) holds advantages over plant cellulose due to its absence of lignin and hemicellulose, resulting in reduced purification expenses and diminished environmental pollution stemming from complex chemical processes. Besides high purity, BC exhibits remarkable characteristics, including high crystallinity (ranging from 70% to 80%), exceptional mechanical attributes, significant water‐holding capacity (up to 100 cc/g), and biological adaptability (Haghighi et al., 2021). A notable advantage of BC is its ability to be readily converted into nanofibrils through mechanical shearing and controlled acid hydrolysis, which makes it a distinctive reinforcement agent within a biopolymer nanocomposite.

The addition of an antimicrobial agent to nanocomposite film has proven to be effective in prolonging the shelf life of foodstuffs and enhancing their quality. Silver nanoparticles are widely used as antimicrobial agents in food packaging due to their distinctive characteristics, which include small particle size, large surface area, and potent antimicrobial properties, all while maintaining low toxicity. Reports indicate that the antibacterial and antifungal efficacy of AgNPs surpasses that of other common metal ions, that is, zinc, gold, and copper (Azizi‐Lalabadi et al., 2021; Kanikireddy et al., 2020). It seems that the inclusion of AgNPs in active films represents a promising strategy for suppressing microbial growth in bread. Furthermore, AgNPs can have a positive impact on the physicomechanical characteristics of the films, a beneficial characteristic for food packaging applications (Rhim et al., 2013).

To the best of our knowledge, no study has been conducted on the effect of CS/BCNF/AgNPs nanocomposite film on bread shelf life and staling. Thus, this research aimed to analyze the optical, thermal, structural, morphological, and water barrier attributes of CS/BCNF films reinforced with varying concentrations of AgNPs and to assess the impact of this active packaging on the physicochemical and microbial characteristics of bread.

## 2 MATERIALS AND METHODS

### 2.1 Materials

Chitosan (70%–85% deacetylated, medium molecular weight, 190–310 kDa) and liquid glycerol were sourced from Sigma Chemical Co. (USA). Silver nanopowder was obtained from FineNano Co. (Iran), while bacterial cellulose nanofiber was purchased from NanoZist polymer Pars Co. (Iran). Microbial media were procured from Merck (Germany). Analytical‐grade chemicals were used for all experiments.

### 2.2 Preparation of films

The preparation of CS/BCNF/AgNPs films was performed according to Salari et al. (2018) method. Briefly, 1 g of chitosan powder was dissolved in 0.1 L of acidic water containing 2% v/v acetic acid. Simultaneously, bacterial cellulose nanofiber (BCNF) was added to distilled water and stirred for 1 h. Then, sonication was done in an ultrasonic bath for 30 min at ambient temperature. A BCNF suspension (4% wt. based on solid matter) and chitosan solution were mixed and stirred for 30 min. Subsequently, various contents of silver nanoparticles (0.5%, 1%, and 2% wt. based on solid matter) were uniformly dispersed in water by an ultrasonic processor at 50% amplitude for 30 min. The silver suspension was then mixed into the solution and stirred for another 30 min. After the addition of glycerol (30%wt. based on solid matter) as a plasticizer, the mixture was stirred for a further 30 min. The resultant emulsions were poured onto plastic petri dishes and dried at 25°C. Conditioning of the films was achieved by storing them in a desiccator with 50% RH for 2 days.

### 2.3 Characterization of active films

#### 2.3.1 Water vapor permeability (WVP)

For WVP evaluation, the film samples were wrapped in the bottle cap containing 3.0 g of anhydrous CaSO_4_ (0% RH). Subsequently, the bottles were stored in a desiccator which contained saturated K_2_SO_4_ (97% RH) solution at 25°C. The weight of the bottles was recorded every 24 h over 7 days. The water vapor transmission rate (WVTR) was obtained as the slope (S) of the weight versus time curve divided by the transfer area (A). Finally, WVP was assessed as follows (Sahraee et al., 2020):

(1) $$\mathrm{WVP}\;\left(g/\text{smPa}\right)=\frac{\text{WVTR}\times d}{∆P}$$

where *d* and *ΔP* are the film thickness (m) and the driving force (Pa), respectively.

#### 2.3.2 Light transmission and opacity

To assess the barrier characteristic of the films against ultraviolet–visible light, a spectrophotometer (DR6000 UV–VIS Laboratory Spectrophotometer—HACH, USA) was employed at 200–800 nm. Films (10 × 40 mm) were directly inserted into glass cuvettes, with an empty cuvette serving as the reference. The film opacity was measured following the method outlined in Putri et al. (2023) using the following Eq:

(2) $$\text{Opacity}={\mathrm{Abs}}_{600}/d$$

where *Abs*_*600*_ and *x* are the values of absorbance at 600 nm and thickness of film (mm), respectively.

#### 2.3.3 Fourier‐transform infrared (FTIR) spectroscopy

FTIR spectrometry was utilized to analyze the structure of films and nanoparticles. Spectra were acquired within the wave number range of 4000–400 cm^−1^ using an FTIR spectrometer (Tensor 27, Bruker, Germany), with 100 scans recorded with 4 cm^−1^ resolutions.

#### 2.3.4 X‐ray diffraction (XRD)

XRD analysis of films and nanoparticles was obtained using a Bruker D5000 X‐ray diffractometer (Siemens, Germany). The equipment was operated at 40 kV and 30 mA using CuKα with 0.154 nm. The analysis was carried out at 25°C within the 2θ = 5°–50°, with an angle step size of 0.05° and rate of 1°/min.

#### 2.3.5 Differential scanning calorimetry (DSC)

The thermal stability of the film was analyzed by DSC instrument (Mettler Toledo, Switzerland). In this regard, the film sample (5.0 mg) was placed in a standard pan, sealed, and scanned at a rate of 10°C/min between 30 and 260°C under a nitrogen atmosphere (20 mL/min). An empty pan was utilized as a reference.

#### 2.3.6 Thermogravimetric analysis (TGA)

Thermal characterization of the films was performed using a TGA instrument (Rheometric Scientific‐STA 1500, United Kingdom). TGA tests were performed in a nitrogen atmosphere (50 cm^3^/min) with a heating rate of 10°C/min. Five milligrams of film sample was used as a standard, and the scanning range was set between 0 and 600°C.

#### 2.3.7 Scanning electron microscopy (SEM)

The surface images of the film samples were characterized by SEM (TESCANMIRA111, Czech Republic) with an accelerator voltage of 26 kV.

### 2.4 Characterization of sliced wheat bread

#### 2.4.1 Preparation of coated bread

To ensure optimal contact between the active agents and bread samples, the bread slices were coated with 50.0 mL of each suspension using a sterilized brush, followed by drying the coatings under ventilation in an aseptic environment. The coating formulations mirrored those of the films mentioned in Section 2.2. Before coating, suspensions were sterilized by autoclaving at 121°C for 20 min.

#### 2.4.2 Water activity (a_w_) and moisture content (MC) of coated bread samples

Water activity of samples was evaluated by an a_w_ meter (ROTRONIC HC2‐AW‐USB, Switzerland) at room temperature. The MC analysis of the coated bread was done by determining the weight loss of samples before and after drying at 110 ± 1°C in a laboratory oven (Shimaz Co., Iran) until a constant weight. Approximately 5.0 g of ground bread was used for each experiment.

#### 2.4.3 Hardness determination

The hardness of the coated bread samples was evaluated by a penetration test conducted with a texture analyzer (KOOPA.TA20, Iran) with a 36 mm measuring probe and operated at a test speed of 0.5 mm/s. The thickness of the bread slices was standardized to 20 mm.

#### 2.4.4 Fungal suspension preparation

*Aspergillus Nige*r (PTCC 5298) was acquired from the Persian Type Culture Collection (Tehran, Iran). The preparation of fungal suspensions was performed using Balaguer et al. (2013) method with some modifications. The fungal suspension at the concentration of 10^6^ CFU/mL was prepared and the Neubauer improved method was used for the counting of spore concentration.

#### 2.4.5 Antifungal properties of nanocomposite films

The preservative‐free wheat bread was bought from a supermarket in Yazd, Iran. Inoculation of the bread slices with *Aspergillus niger* suspension was done following the method reported by Balaguer et al. (2013). Bread samples were inoculated with the *Aspergillus niger* suspension (5 μL) at three points. To examine the active films' effect against naturally occurring fungi in bread, some slices were left noninoculated. All samples (both inoculated and non‐inoculated) were packed with nanocomposite films and placed in polyethylene bags (25 × 15 cm) and then assessed visually for fungal growth over 60 days at 25°C. Control samples were prepared similarly but without any active nanocomposite film.

#### 2.4.6 Antifungal properties of active coatings

The coated bread samples, prepared as Section 2.4.1, were packaged in polyethylene bags (25 × 15 cm) and kept at room temperature (25°C) for 15 days. Bread slices weighing 10.0 g were aseptically and randomly sampled, poured into a flask that contained 0.9 L physiological saline solution (0.9% NaCl w/v), and homogenized for 6 min in a mixer at 260 rpm. Subsequently, dilution series (10^−1^–10^−5^) were prepared. The fungi and mold numbers were evaluated using potato dextrose agar (PDA) incubated at 25°C for 5 days and expressed as log CFU per gram of bread (log CFU/g) (Noshirvani et al., 2017).

### 2.5 Statistical analysis

Each analysis was done three times in a completely randomized design. Statistical analysis was done using the analysis of variance (ANOVA) procedure, employing a completely randomized design, with SPSS 24 (SPSS Inc., USA). Differences among means at the level of 5% were evaluated by Duncan's multiple range tests.

## 3 RESULT AND DISCUSSIONS

### 3.1 Active film characterization

#### 3.1.1 WVP

As the reduction in WVP is crucial for improving the shelf life of packed food products, films with low WVP values are deemed ideal as food packaging materials. The WVP of the films was remarkably enhanced by the incorporation of BCNF and AgNPs into the film matrix (Table 1). The notable difference in WVP between the control and CS/BCNF films is primarily attributed to increased tortuosity and crystallinity of the film, along with the hydrogen bond formation between BCNF and chitosan in the prepared films. These factors contribute to preventing the formation of pores through which water molecules could permeate. The obtained results are in line with Haghighi et al. (2021). In addition, the impermeable crystal structure of BCNF leads to a decrease in the water transport rate.

**TABLE 1:** Water vapor permeability (WVP), light transmission percentage, and opacity of chitosan and nanocomposite films.

Samples	WVP (×10^−10^ g/smPa)	Light transmission (%)			Opacity
		280	600	800	
Chitosan	3.75 ± 0.07^a^	8.58 ± 0.83^a^	71.30 ± 1.08^a^	80.57 ± 1.65^a^	3.02 ± 0.11^d^
CS/BCNF	2.58 ± 0.23^b^	6.56 ± 0.67^b^	60.50 ± 0.72^b^	76.74 ± 1.08^b^	4.78 ± 0.13^c^
CS/BCNF/0.5% AgNPs	1.95 ± 0.1^c^	4.94 ± 0.06^c^	48.54 ± 1.69^c^	71.75 ± 1.78^c^	5.22 ± 0.19^c^
CS/BCNF/1% AgNPs	1.59 ± 0.17^d^	4.01 ± 0.27^d^	45.36 ± 0.53^d^	66.25 ± 0.92^d^	6.03 ± 0.55^b^
CS/BCNF/2% AgNPs	0.85 ± 0.09^e^	2.59 ± 0.21^e^	32.19 ± 2.45^e^	50.21 ± 0.12^e^	6.77 ± 0.47^a^

*Note*: Means within each column with the same letter are not significantly different (*p* < .05). Data are mean ± SD.

The WVP of CS/BCNF film decreased with increasing AgNPs content, and the minimum WVP of 0.85 × 10^−10^ g/smPa was obtained for CS/BCNf/2% AgNPs (Table 1). This could be attributed to the fact that impermeable AgNPs in the polymer matrix increase the tortuous pass‐way of the crossing water vapor, which decreases permeability. The presence of nanoparticles decreases the mobility of polymer chains, thus improving the barrier properties (Kanmani & Rhim, 2014). Besides, active agents can block the film network pores, thereby preventing the penetration of water molecules (Mihaly‐Cozmuta et al., 2017). Finally, the incorporation of BCNF and AgNPs can improve the nanocomposite film resistance against water.

#### 3.1.2 Light transmission and transparency

Light transmission of active films across the wavelength at 280, 600, and 800 nm was analyzed (Table 1). The presence of AgNPs and BCNF in the chitosan film formulation notably decreased ultraviolet and visible light transmission. Thus, the CS/BCNF/2% AgNPs film able to absorb ultraviolet–visible light would have potential application in delaying food oxidative deterioration and help prevent issues such as discoloration, off‐flavors, and nutrient losses. This phenomenon is primarily attributed to the scattering of light and prevention of light passage by homogeneously dispersed nanoparticles in the polymer matrix. The results obtained are in line with those described by Kanmani and Rhim (2014) and Sahraee, Ghanbarzadeh, et al. (2017)for the impact of AgNPs and N‐chitin on the transparency of gelatin films, respectively.

The opacity is an indication of film transparency. The greater film transparency leads to lower opacity. As can be observed in Table 1, the CS film exhibited a transparent appearance because of its relatively low opacity (3.02). However, upon the addition of BCNF and AgNPs, the opacity of the CS/BCNF and CS/BCNF/2% AgNPs nanocomposite films showed higher values of 4.78 and 6.77, respectively. This increase in opacity is attributed to the incorporation of AgNPs as an inorganic substance that does not dissolve in the chitosan film (Arfat et al., 2014).

#### 3.1.3 FTIR

**FIGURE 1:**
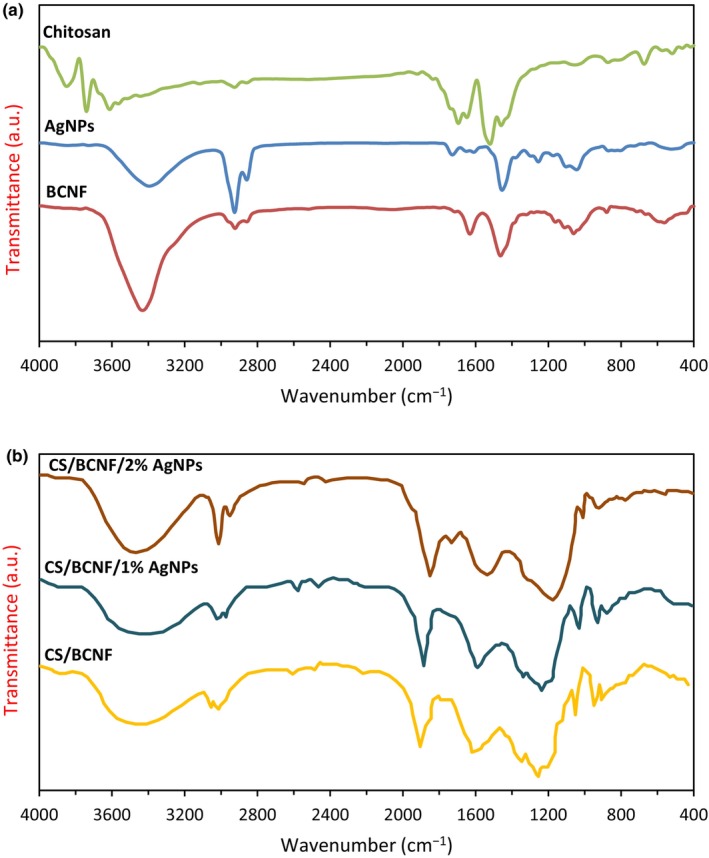
FTIR spectra of chitosan, BCNF and AgNPs (a), and nanocomposite films (b) (in color).

FTIR spectroscopy is a useful technique to investigate chemical bonds in the structures of films. Figure 1a,b displays the FTIR spectra of film samples. In the case of chitosan, the peak at 1648 was related to C=O stretching in amide I, and 1520 cm^−1^ was assigned to N‐H bending in amide II. The broadband above 3000 cm^−1^ is assigned to the O‐H and N‐H stretching bonds. The peak that appeared at 1050 cm^−1^ is due to C–O bond stretching, while the amino group appeared at 2900 cm^−1^. The assignment of these absorption bands is in line with those obtained by previous studies (Kongkaoroptham et al., 2021; Leceta et al., 2013).

In the FTIR spectrum of BCNF, the absorption band observed from 3600 to 3200 cm^−1^ is typically related to stretching vibrations of O‐H. The typical sharpening at 3335 cm^−1^ might be due to intramolecular hydrogen bonding. The peaks around 3000–2800 cm^−1^ are attributed to CH stretching vibrations of CH_2_ and CH_3_ groups. The significant peak at 1160 cm^−1^ is related to stretching vibrations C‐O‐C, while the bands at 1060 cm^−1^ and 1035 cm^−1^ correspond to the C–O bonds of aliphatic secondary and primary alcohols of cellulose, respectively (Haghighi et al., 2021).

Compared to BCNF and the chitosan film, the bonds at 3200–3450 cm^−1^ of the nanocomposite films shifted and became more intense with the incorporation of nanocellulose, indicating the creation of hydrogen bonding between the BCNF and CS (Y. Bao et al., 2018). The FTIR spectra of CS/BCNF/AgNPs nanocomposite films showed no additional peak formation, except the peak height, which indicated that no appreciable chemical interaction occurred between CS and AgNPs.

#### 3.1.4 XRD

**FIGURE 2:**
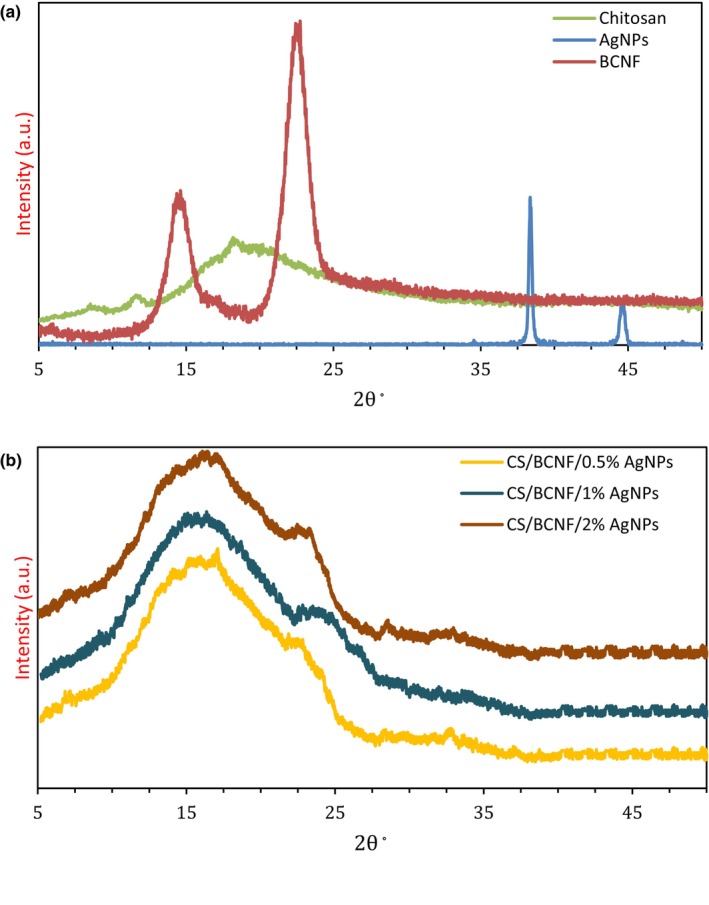
XRD patterns of chitosan, BCNF and AgNPs (a), and nanocomposite films (b) (in color).

Figure 2a,b illustrates the XRD patterns of BCNF, AgNPs, and film samples. The diffraction peaks of BCNF are shown at 14.64°, 16.74°, and 22.78°, attributed to (101), (101), and (002), crystalline planes of cellulose I, respectively (Li et al., 2009; Nam et al., 2021). Film samples demonstrated prominent peaks at 2θ = 11° and a relatively broad diffraction peak at around 2θ = 20°, which depict their hydrated crystalline structure and semiamorphous nature, respectively (Kongkaoroptham et al., 2021).

Due to BCNF addition, the broad peak of semiamorphous chitosan at 2θ = 20° overlaid with the intense peak of BCNF at 22.7°. This phenomenon demonstrated that CS/BCNF film represented a combination of crystalline and amorphous peaks. The peak intensity increased by BCNF addition into the chitosan film. The findings indicate that the BCNF addition in CS film did not notably alter the position of specific characteristic bands in the XRD spectrum. This suggests an increase in crystallinity rather than a change in crystal configuration in the CS/BCNF nanocomposite film. The same findings were obtained by Sun et al. (2018), who reported that nanocrystalline cellulose (NCC) addition into a polymer matrix can promote crystallization by reducing of free energy barrier.

The distinctive diffraction peaks at 38.1 and 44.2 were related to (111) and (200) planes of silver, respectively, confirming the formation of a face‐centered cubic (fcc) structure of the crystalline silver nanoparticles (Kanikireddy et al., 2020). It was quite clear that the number of diffraction peaks and peak height at specific diffraction positions increased with an increase in the content of AgNPs. These findings show that the CS, BCNF, and AgNPs have excellent compatibility.

#### 3.1.5 DSC

**FIGURE 3:**
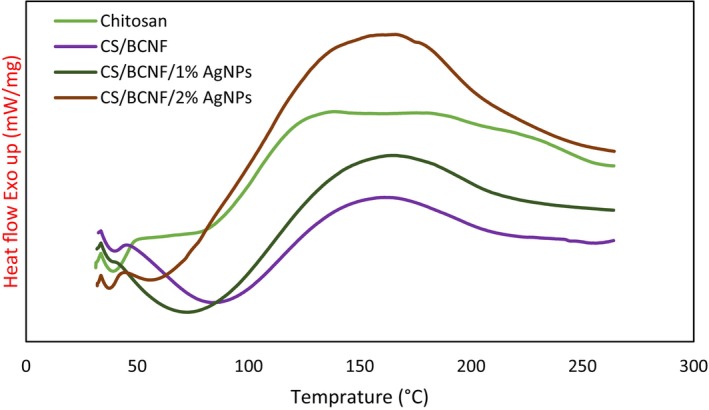
DSC thermograms of chitosan, and nanocomposite films (in color).

The T_m_ and ΔH_m_ of film samples are detailed in Table 2, and their DSC curves are shown in Figure 3. As can be seen, the addition of BCNF and AgNPs in chitosan films led to a notable enhancement in their thermal properties. The melting point and enthalpy of fusion of the film samples were enhanced upon the incorporation of BCNF and AgNPs. This suggests that nanoparticles can enhance the crystalline behavior of chitosan film. These findings are in line with XRD analysis. Similar behaviors were observed in previous studies (George & Siddaramaiah, 2012; Reiad et al., 2013). Meera and Ramesan (2023b) stated that the strong interaction of the polar boehmite nanoparticles with the carboxymethyl chitosan/cashew gum macromolecular chain leads to an increase in the compactness of the blend film and, as a result, an increase in the melting temperature. Similarly, it was revealed that by reinforcing of chitosan matrix with hydroxyapatite nanoparticles (HANPs) 1%, the T_m_ of film shifted to higher temperature (Upadhyay & Ullah, 2024). Moreover, the result reveals a significant transition in the values of ΔH_m_ with an enhancement in AgNPs content. This occurrence may be credited to nanoparticles enhancing the polymer's overall crystallinity, facilitating the development of compressed and orderly crystals. Consequently, additional energy is necessary to disturb the heightened crystalline arrangement; their incorporation into film formulation can increase the heat stability of obtained nanocomposite films (Kadam et al., 2019; Sahraee, Ghanbarzadeh, et al., 2017).

**TABLE 2:** T_m_ and ΔH_m_ of chitosan and nanocomposite films.

Samples	T_m_ (°C)	ΔH_m_ (J/g)
Chitosan	138.96	28.54
CS/ BCNF	159.16	148.31
CS/BCNF/1% AgNPs	164.27	156.08
CS/BCNF/2% AgNPs	169.12	188.43

#### 3.1.6 TGA

**FIGURE 4:**
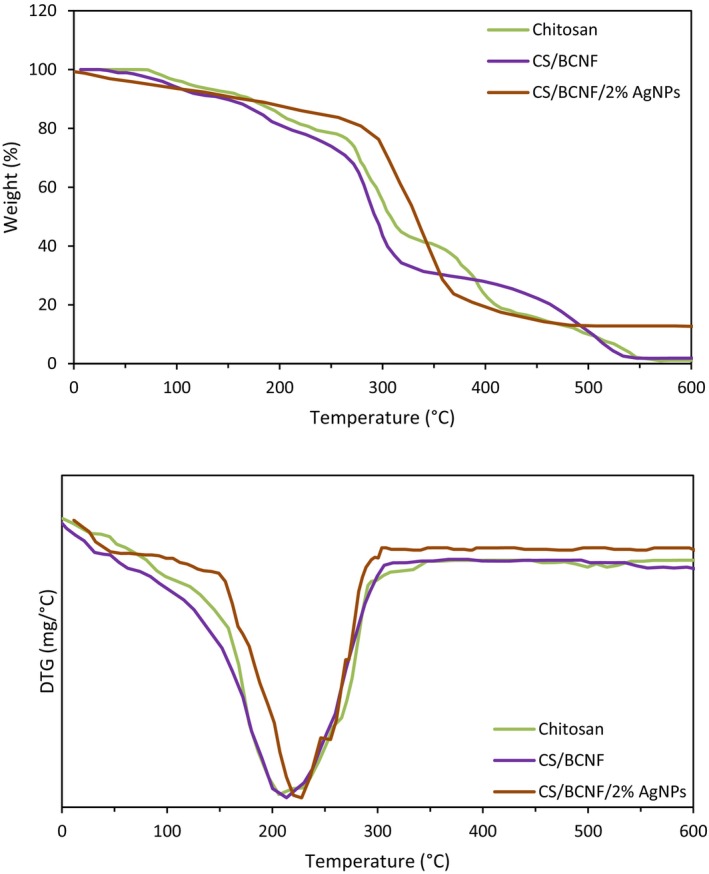
TGA and DTG thermograms of chitosan, and nanocomposite films. (in color).

Thermogravimetric analysis (TGA) and resulting thermogravimetric (DTG) curves were used to explore the effectiveness of employed nanoparticles on the thermal decomposition of the nanocomposite films. For all film samples, thermal analysis showed a multistage degradation pattern (Figure 4a,b). The first step, occurring around 100°C, was linked to moisture evaporation and residual acetic acid. Stage II, recorded at 250–350°C, is because of the decomposition side chains of CS and BCNF, and the decomposition of glycerol at the same time. Phase III observed at 420–600°C may arise from the oxidative degradation of the carbonaceous residue generated in Phase II (Nouri et al., 2018). It was observed that the onset of thermal degradation for AgNPs (2%) loaded CS/BCNF film was delayed slightly, indicating an enhancement in the thermal resistance because of more heat‐stable metallic silver (Rhim et al., 2013). The variance in weight reduction between the chitosan film and nanocomposite primarily stems from the addition of AgNPs into the formulated chitosan nanocomposite. The DTG peak implied (Figure 4b) that the peak maximum temperature of chitosan, CS/BCNF, and CS/BCNF/2% AgNPs was observed at 206.13, 213.24, and 227.44°C, respectively. From this finding, it is found that the thermal stability of chitosan film is enhanced after nanocomposite preparation by BCNF and AgNPs. A similar result was reported earlier (Perumal et al., 2018; Thiagamani et al., 2019). According to our findings, Meera and Ramesan (2023a) demonstrated that by increasing the content of nanofiller (boehmite nanoparticles) in the carboxymethyl chitosan/polyvinyl alcohol blend film, the thermal stability increases. Nanoparticles typically function as fillers, providing heat insulation and enhancing the interaction between polymer chains. They also act as a barrier against volatile compounds resulting from polymer decomposition, and can increase the thermal properties of polymers (Sahraee, Milani, Ghanbarzadeh, & Hamishehkar, 2017b).

#### 3.1.7 Film microstructure

**FIGURE 5:**
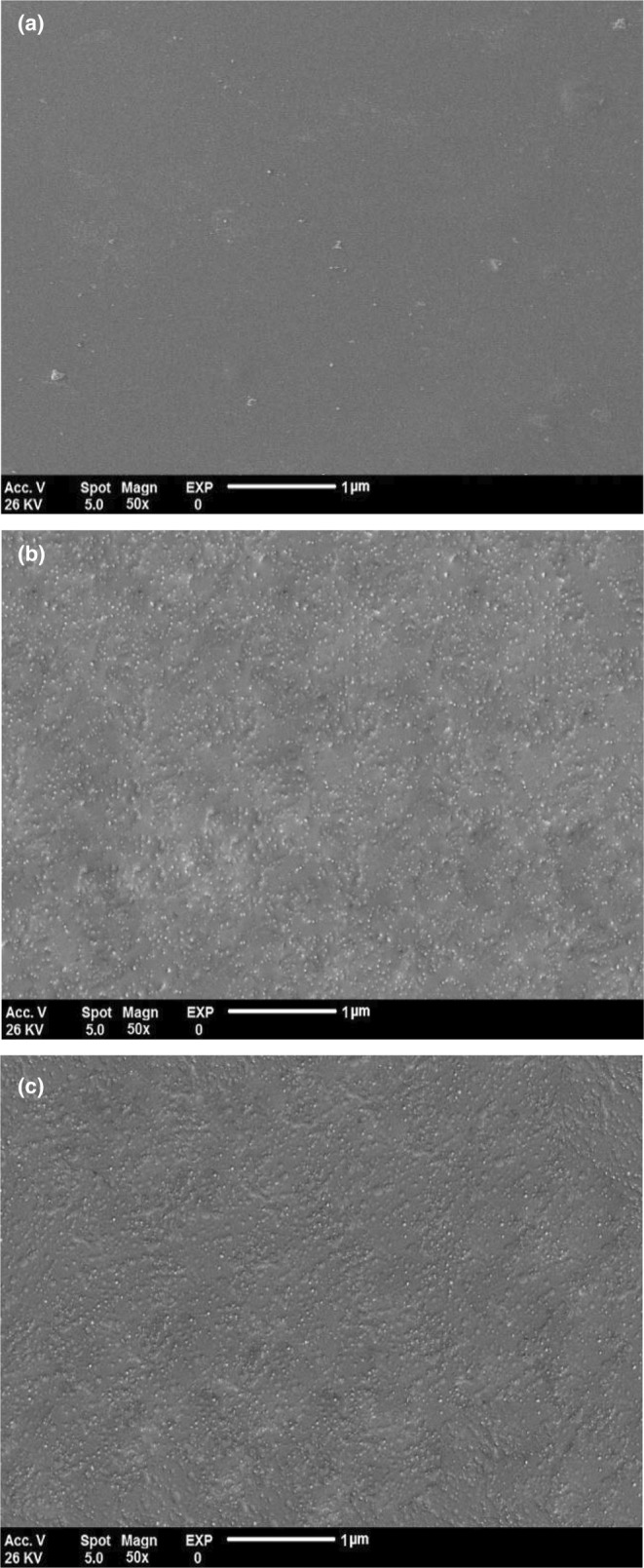
SEM micrographs of chitosan (a), CS/BCNF/1% AgNPs (b), and CS/BCNF/2% AgNPs (c).

Figure 5a–c displayed the visual macroscopic images of chitosan, CS/BCNF/1% AgNPs, and CS/BCNF/2% AgNPs. The microstructure of the chitosan film revealed a flat, uniform, and smooth surface, suggesting improved homogenization of chitosan in aqueous medium. The SEM image of CS/BCNF/1% AgNPs film is characterized by a dense and homogeneous structure with uniformly distributed BCNF and AgNPs and cellulose nanofibers illustrated like white dots in the main polymeric film. It confirmed the excellent compatibility and interaction of the nanoparticles with the CS, which enhances the physical and thermal properties of nanocomposites. However, with increasing AgNPs content, the film surface became rougher, and some aggregation or bigger size particles were observed (Figure 5c).

### 3.2 Wheat bread characterization

#### 3.2.1 Water activity (a_w_) and moisture content (MC)

**FIGURE 6:**
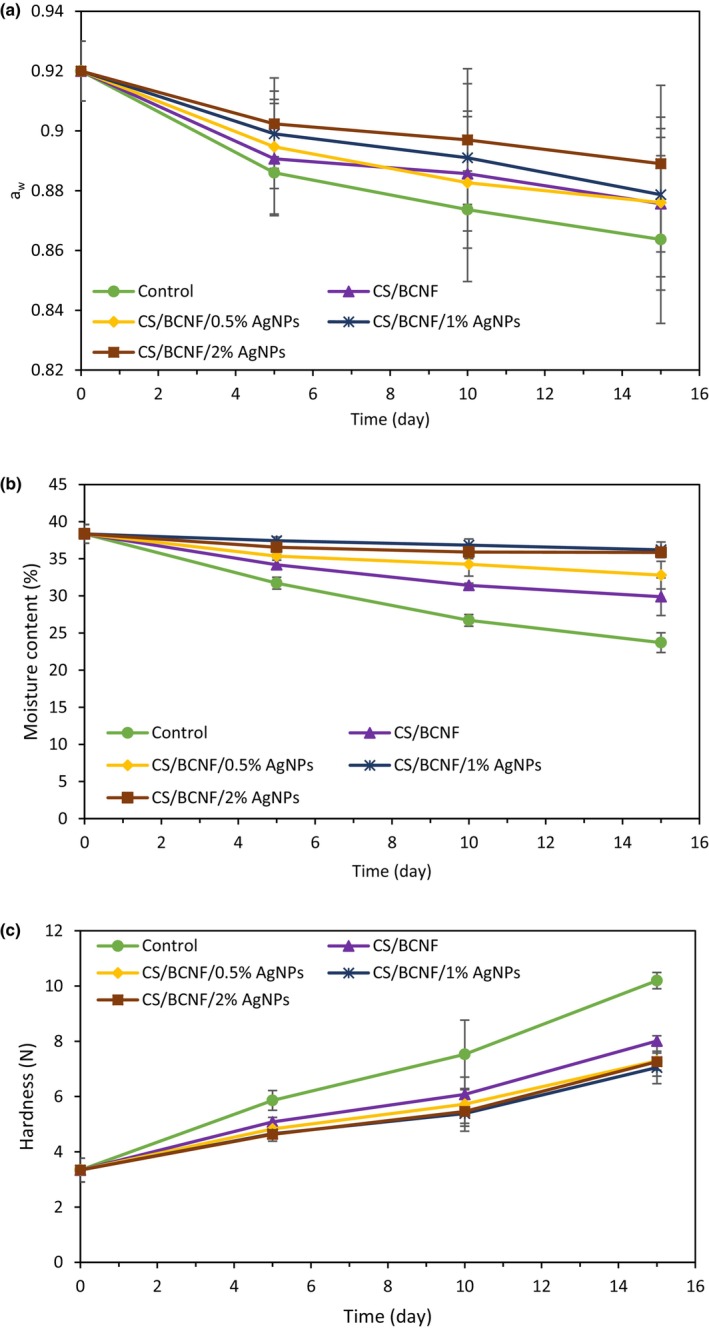
a_w_ (a), moisture content (b), and Hardness (c) of coated bread samples stored for 15 days. Control was uncoated bread (in color).

The water activity and moisture content of the coated bread samples were determined during storage over 15 days (Figure 6a,b). Results showed that both a_w_ and MC decreases during the time were various, influenced by coating formulations; however, the maximum reduction was detected for control (bread without coating) compared to the other samples. The active coating's presence might aid in retaining water within the bread by limiting moisture migration from the bread crust to the surrounding environment. Considering the impact of moisture content on the freshness of bread, preserving water within the bread could enhance bread properties and mitigate staling. As mentioned in Section 3.1.1., the addition of BCNF and AgNPs lowered the WVP of the CS film, which is in accordance with the results of a_w_ and MC. In addition, the increasing presence of AgNPs could enhance moisture preservation in bread due to the hygroscopic properties of silver nanoparticles and their positive impact on improving water vapor barrier properties, as previously discussed. Similar findings were conducted by Jafarzadeh et al. (2021), who reported that further mitigate weight loss by serving edible coatings as a barrier against moisture loss.

#### 3.2.2 Hardness

As illustrated in Figure 6c, the hardness of the crumb gradually decreased throughout storage for all samples. This is mainly attributed to the water loss because of the difference in vapor pressure between bread crumbs and the surrounding headspace, which promotes hardening. Besides, the recrystallization of amorphous materials, particularly starch, accelerated the release of adsorbed water and enhanced hardness. The highest hardness value belonged to the control sample. In coated bread samples, lower hardness was observed for bread samples covered with CS/BCNF/AgNPs, followed by the CS/BCNF and chitosan films, respectively. The hardness results demonstrate a notable impact of BCNF and AgNPs in reducing water migration across the coatings, consistent with the findings of WVP, a_w_, and, MC tests. The rise in crumb hardness and bread staleness mainly stemmed from moisture transfer from the crumb to the crust, along with starch recrystallization and aggregation after cooling (Melini & Melini, 2018).

#### 3.2.3 Evaluation of fungal growth in inoculated breads

As the growth of surface molds and fungi is the most important factor for the deterioration of wheat products, applying an active nanocomposite is a good strategy to prolong their shelf life. The development of fungal growth on the bread samples, whether packed with or without the active nanocomposite films, is detailed in Table 3. Given that the dough formulation lacked preservatives and organic flour was used, mold growth on the control bread sample was only observed on the third day of storage. Bread packaging by active films proved very effective in retarding fungal development and for CS/BCNF/2% AgNPs, fungal growth appeared on the 28th day of storage (Table 3). Similarly, in a study conducted on inoculated breads with *Aspergillus niger*, it was found that the use of carboxymethyl cellulose‐chitosan‐oleic acid‐ZnO NPs 2% film can increase the shelf life of the samples from 3 to 22 days compared to the control sample without film (Noshirvani et al., 2017).

**TABLE 3:** Assessment of visual observation of inoculated bread samples by *Aspergillus niger* stored for 30 days at 25°C.

Packaging type	Growth delay (days)	Fungal growth intensity			
		Day 5	Day 10	Day 15	Day 30
Control	4	+	++	+++	+++
CS/BCNF	6	−	+	++	+++
CS/BCNF/0.5% AgNPs	13	−	−	+	++
CS/BCNF/1% AgNPs	17	−	−	−	+
CS/BCNF/2% AgNPs	25	−	−	−	+

*Note*: − absence of fungal growth, + fungal growth < 25% of surface, ++ fungal growth ≅ 25–50% of surface, +++ fungal growth >75% of surface.

#### 3.2.4 Evaluation of fungal growth in noninoculated bread

**FIGURE 7:**
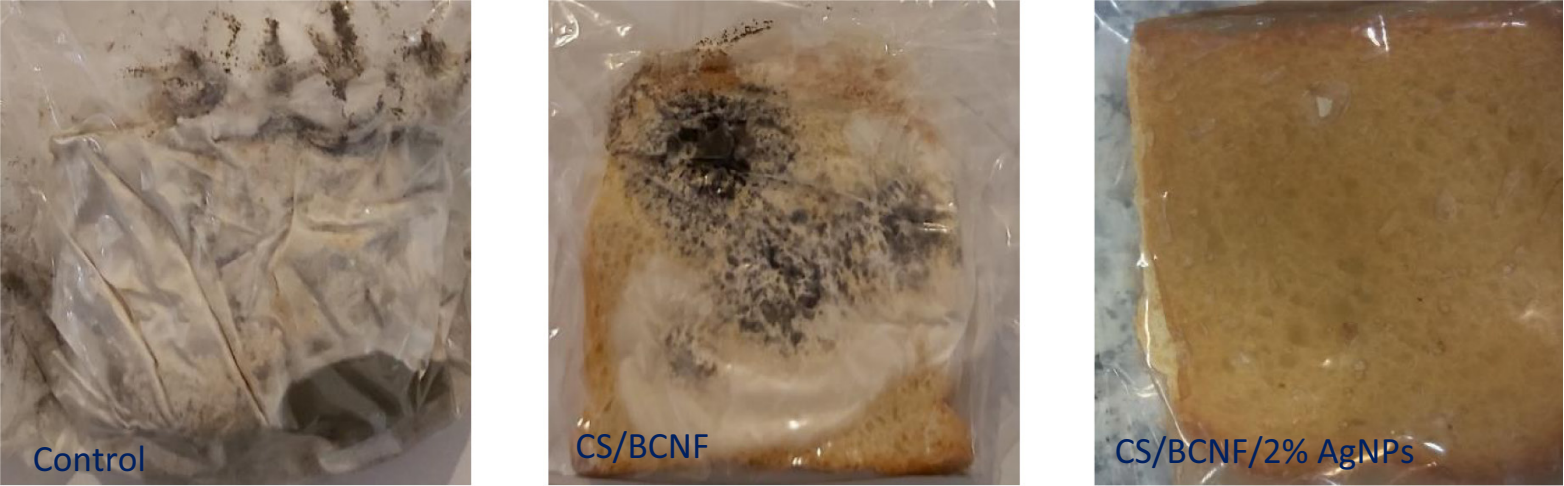
Visual observation of noninoculated bread samples stored for 30 days at 25°C (in color).

To identify the impact of the nanocomposite films against the natural fungi in bread samples, bread slices were wrapped with different active film samples and kept at room temperature until the appearance of fungi growth (Table 4). Figure 7 shows the condition of bread samples attributed to 30 days after storage at 25°C. The growth of fungi in the control sample (bread without film) and bread packed with CS/BCNF was seen at 5 and 8 days of incubation, respectively. The obtained data suggested that CS/BCNF without AgNPs significantly prolonged the shelf life of bread slices compared to bread without any film. This is linked to the antimicrobial attributes of CS, which involve the interaction between microbial and fungal membranes with a negative charge and the amine groups in CS biopolymer with a positive charge that causes the leakage of cell materials and the death of microorganisms. Besides, chitosan, by its chelating properties, can bind with metals, inhibit different microbial cell enzymes, and finally reduce the growth of microorganisms (Kumar et al., 2020). These findings showed that the growth of fungi was inhibited considerably by the incorporation of AgNPs into the CS/BCNF film, which was attributed to the presence of AgNPs in the film formulation. Although the degree of antifungal activity of the nanocomposite films varied depending on the silver content and in bread packed with CS/BCNF/2% AgNPs films, fungal growth was seen on the 38th day of incubation at 25°C. Several mechanistic mechanisms have been reported for the antimicrobial activity of AgNPs. The main mechanism is attributed to the binding of silver ions (positive charge) with biomacromolecular components (negative charge), which leads to protein denaturation, structural destruction, and prevention of DNA replication, followed by the death of the microorganism. Additionally, reactive oxygen species generated by nanoparticles could damage the membrane, leading to cell death. The antimicrobial activity of AgNPs is also affected by the amount of Ag ions released from the silver nanoparticles, which act as reservoirs for Ag ions (Nwabor et al., 2020; Rhim et al., 2013). In several articles, the increase of the microbial shelf life of bakery products by active packaging has been shown. Suwanamornlert et al. (2020) demonstrated an extension in the bread shelf life of bread packaged with PLA/PBSA blend film incorporated with thymol from 3 days for control film to 9 days for active film at 25°C. In addition, Al‐Tayyar et al. (2020) showed the shelf life improvement and microbiological safety of bread by poly (vinyl alcohol)/chitosan/ZnO‐SiO_2_ bio nanocomposites. In the study conducted by Sahraee et al. (2020), it was shown that the use of gelatin film containing antifungal nanoparticles (nano chitin and nano ZnO) can extend the shelf life of packaged sponge cakes up to 28 days. Hosseiniyeh et al. (2023) showed that by using lecithin‐emulsified black seed oil (BSO) nanoemulsions (LNEO) and whey protein isolate‐stabilized Pickering emulsions (WPEO) in the soy protein isolate (SPI)‐based films, the shelf life of bread can be increased up to 17 and 13 days. However, mold growth was observed only after 9 and 6 days of storage in bread samples packed with pure film and low‐density polyethylene. The longer shelf life obtained in this study indicates the very effective antifungal properties of silver nanoparticles, which have been proven before (Kim et al., 2009).

**TABLE 4:** Assessment of visual appearance of fungal growth in bread samples packed in nanocomposite films at 25°C.

Packaging type	Growth delay (days)	Fungal growth intensity			
		Day 5	Day 10	Day 15	Day 30
Control	5	+	++	+++	+++
CS/BCNF	8	−	+	++	+++
CS/BCNF/0.5% AgNPs	18	−	−	−	++
CS/BCNF/1% AgNPs	26	−	−	−	+
CS/BCNF/2% AgNPs	38	−	−	−	−

*Note*: − absence of fungal growth, + fungal growth < 25% of surface, ++ fungal growth ≅ 25–50% of surface, +++ fungal growth >75% of surface.

#### 3.2.5 Antifungal effectiveness of coatings

Table 5 shows the yeasts and mold count for coated bread throughout the storage period. As expected, the yeast and mold numbers in coated bread samples increased as a function of test duration, but this increase in bread slices with active coating was significantly lower compared to the control one. It emphasizes that the active coating has a strong antifungal activity. After a storage period of 15 days, the most favorable outcomes were observed for nanocomposite coatings containing 1% and 2% AgNPs, where fungal growth was not detected. The finding proved the application of CS/BCNF/2% AgNPs film was effective for controlling fungal populations and extending bread shelf life.

**TABLE 5:** Yeast and mold counts (log CFU/g) of coated bread samples at 25°C.

Days	Control	CS/BCNF	CS/BCNF/0.5% AgNPs	CS/BCNF/1% AgNPs	CS/BCNF/2% AgNPs
1	2.01 ± 0.8^d^	1.6 ± 0.04^d^	0^b^	0^a^	0^a^
5	4.71 ± 0.03^c^	2.98 ± 0.11^c^	0^b^	0^a^	0^a^
10	6.84 ± 0.16^b^	4.98 ± 0.08^b^	0^b^	0^a^	0^a^
15	7.91 ± 0.08^a^	5.87 ± 0.12^a^	1.35 ± 0.13^a^	0^a^	0^a^

*Note*: Means within each column with the same letter are not significantly different (*p* < .05). Data are mean ± SD.

## 4 CONCLUSIONS

The primary challenges affecting bread quality are staling and microbial growth, leading to significant economic losses worldwide. To address this fact, chitosan film containing BCNF and/or varying concentrations of AgNPs (0%, 0.5%, 1%, and 2%) was developed to enhance microbial safety and improve bread shelf life. The presence of BCNF and AgNPs in the biopolymer matrix was confirmed through FTIR, XRD, and SEM analyses. Nanocomposite films showed notably improved ultraviolet barrier properties, WVP as well as thermal stability. Based on a_w_ and moisture content results, the coated bread demonstrated superior moisture retention. The inclusion of Ag nanoparticles enhanced the bioactivity of the nanocomposite films and coatings, displaying effective antimicrobial activity against fungi. In summary, the CS/BCNF/2% AgNPs nanocomposite films represent promising packaging films for prolonging the bread shelf life and potentially various food products. Additionally, CS/BCNF/2% AgNPs coating can mitigate the rate of bread staling. However, further research should be done to evaluate the effect of active packaging containing AgNPs and BCNF on the sensory properties of foodstuffs, as well as the migration rate of silver nanoparticles to these products.

## AUTHOR CONTRIBUTIONS

**Jalal Sadeghizadeh Yazdi:** Funding acquisition (equal); project administration (equal). **Mahdieh Salari:** Formal analysis (equal); methodology (equal); software (equal); writing – original draft (equal). **Mohammad Hasan Ehrampoush:** Funding acquisition (equal). **Mehrasa Bakouei:** Methodology (equal).

## CONFLICT OF INTEREST STATEMENT

The authors declare no conflict of interest.

## Data Availability

All the data used are shown in this article.
